# Ego Depletion in Real-Time: An Examination of the Sequential-Task Paradigm

**DOI:** 10.3389/fpsyg.2017.01672

**Published:** 2017-09-26

**Authors:** Madeleine M. Arber, Michael J. Ireland, Roy Feger, Jessica Marrington, Joshua Tehan, Gerald Tehan

**Affiliations:** School of Psychology and Counselling and Institute for Resilient Regions, University of Southern Queensland, Springfield, QLD, Australia

**Keywords:** ego depletion, strength model, self-regulation, sequential task, letter crossing

## Abstract

Current research into self-control that is based on the sequential task methodology is currently at an impasse. The sequential task methodology involves completing a task that is designed to tax self-control resources which in turn has carry-over effects on a second, unrelated task. The current impasse is in large part due to the lack of empirical research that tests explicit assumptions regarding the initial task. Five studies test one key, untested assumption underpinning strength (finite resource) models of self-regulation: Performance will decline over time on a task that depletes self-regulatory resources. In the aftermath of high profile replication failures using a popular letter-crossing task and subsequent criticisms of that task, the current studies examined whether depletion effects would occur in real time using letter-crossing tasks that did not invoke habit-forming and breaking, and whether these effects were moderated by administration type (paper and pencil vs. computer administration). Sample makeup and sizes as well as response formats were also varied across the studies. The five studies yielded a clear and consistent pattern of increasing performance deficits (errors) as a function of time spent on task with generally large effects and in the fifth study the strength of negative transfer effects to a working memory task were related to individual differences in depletion. These results demonstrate that some form of depletion is occurring on letter-crossing tasks though whether an internal regulatory resource reservoir or some other factor is changing across time remains an important question for future research.

## Introduction

Self-regulation refers to dynamic efforts to monitor and adapt behavior, attention, emotions, and cognitive strategies in a goal-directed way (Carver and Scheier, 2000). The focus of this paper is concerned with one theory of self-regulation, but is aimed at addressing some of the current “crises” besetting self-regulation research. We will first outline the theory and methodology employed to test the theory and then examine what have been labeled the “replication crisis” in psychology (Pashler and Harris, 2012) and the “conceptual crisis” associated with self-regulation research in particular (Lurquin and Miyake, 2017).

While there are a number of different theories that address self-regulation, some based upon shifts in motivation (Inzlicht and Schmeichel, 2012), and some based on notions of cognitive control (Dang et al., 2017), the current experiments deal specifically with the strength model of self-regulation which still exerts influence on current research in spite of doubts concerning the veracity or utility of the model (e.g., Inzlicht and Berkman, 2015). While we focus on this model, we would argue that the issues raised have implications for other models as well.

The strength theory of “ego depletion” is an account of processes believed to underlie self-regulation, and importantly, explain regulatory failures (Baumeister et al., 1998, 2000). The ego depletion framework posits a strength-model or resource-model of self-regulation, whereby the ability to execute regulatory functions mirrors the familiar processes of a muscle temporarily fatiguing with use. A finite supply of internal psychological resources is hypothesized to be available to support regulatory actions and these resources are “spent” in the act of performing them. More precisely, the capacity to carry out the higher-order executive functions that underpin self-regulation and self-control (e.g., concentration and attention regulation, impulse control, emotion regulation, and behavioral inhibition) is governed by the availability of a finite internal psychological resource. Ego depletion refers to a state in which internal resources have become diminished, executive function capacity is reduced, and the likelihood of self-regulatory failure is enhanced (Baumeister and Alquist, 2009).

In the laboratory, ego depletion effects are typically investigated using the “sequential-task paradigm” (or “dual-task” paradigm). As the name suggests, this experimental paradigm involves testing for performance deficits on the second of two tasks (the outcome task) that result from completing an initial task designed to tax self-regulation resources (the depletion task). Participants' performance following a depletion task is then compared to control subjects that have not spent resources on the initial depleting task, with the expectation that those in the experimental group will show poorer performance on the outcome task than the control group. From the perspective of the strength model, two key assumptions underpin the use and interpretations of the dual-task paradigm: firstly, that engagement in the depleting task consumes self-regulatory resources; and secondly, that the decline of self-regulation resources causes the observable deficits on a subsequent self-regulatory task. Many studies have tested and provided supporting evidence for the predictions of the dual-task paradigm (see Hagger et al., 2010 for a review); however this line of evidence has exclusively focused on measuring group differences in carry-over effects on the second task without scrutinizing the actual changes in performance occurring within the depleting task itself.

## “Replication crisis”

In spite of a coherent body of confirmatory evidence, including meta-analyses, suggesting that depletion effects are reliable and moderately sized [*d* = 0.62, (95% CI: 0.57, 0.67)], more recent meta-analyses have cast doubt on the true magnitude of these effects. Carter and McCullough (2014) suggested that the effect size might be an over-estimate of the true size given publication biases to positive results and the increased likelihood of obtaining such positive effects in experiments that have small numbers of participants. In a subsequent meta-analysis Carter et al. (2015) examined effect size as a function of the outcome task used, showing that carry-over depletion effects differed across tasks, but again, when bias-correction techniques were adopted, effect sizes were not distinguishable from zero. Moreover, the most recent meta-analysis focused solely on the Stroop Task and found little evidence to support the strength model, and what evidence there was, was contaminated by publication bias (Dang et al., 2017).

Just as the meta-analyses cast doubt on the veracity of ego-depletion, there are now a number of highly publicized failures to replicate the phenomenon (Xu et al., 2014) or found it to be substantially smaller in size than reported in meta-analytic syntheses (Tuk et al., 2015). In one large large-N, multi-site study involving 23 different laboratories across English speaking and non-English speaking countries (Hagger and Chatzisarantis, 2016) the same protocol was administered, consisting of a letter crossing manipulation task and the Multi-Source Interference Task as the outcome task. Of the 23 replications approximately half produced positive outcomes and half produced negative outcomes of differing strengths. Overall the small positive effect could not be distinguished from zero. Thus, corrections for small study and publication biases plus failures to replicate question whether ego-depletion is a real phenomenon (Hagger and Chatzisarantis, 2016; Lurquin et al., 2016).

## “Conceptual crisis”

While the meta-analysis and failures to replicate suggest that the strength model has been largely discredited, Lurquin and Miyake (2017) have argued that the ego depletion literature as a whole suffers from a conceptual crisis as well as a replication crisis. They argue that there is a lack of clear operational definitions of self-control; a lack of independent empirical validation for self-control tasks; and a lack of well-specified models that make unambiguous, falsifiable predictions. The lack of independent validation of self-control tasks is readily seen in Baumeister and Vohs (2016) response to the failed multi-site replication experiment. They argued that depletion only occurs under a limited set of task parameters. The depletion task must first set up a habitual response, and then change the task requirements such that this habitual response must be resisted. They argued that failures in replication resulted from an absence of first creating a habitual response. The depletion task could not (rather than failed to) induce ego depletion. A second reason provided for the failed multi-site replication was the assertion that the use of computer-administered tasks was sub-optimal for inducing depletion and that pencil and paper administrations of the depletion task were more potent manipulations. These are not the only parameters that have been proposed to explain different outcomes. The time on task and the level of difficulty have often been used as *post-hoc* explanations for observed patterns of performance. Moreover, Dang et al. (2013), in one of the few studies that has explored performance on the depletion task across time, raise the interesting possibility that with extended time on the depletion task participants can adapt to the task (in this instance a Stroop task) and “replenish” resources. This later study questions one other fundamental assumption of the strength model that resource depletion occurs over time.

While debate continues about what tasks are truly depleting and why, these events highlight some fundamental considerations about the nature and effect of the depleting task itself. What happens in the depletion task is crucial, as changes in performance, or lack of them, can falsify theories, constrain them or provide confirming evidence for one and disconfirming evidence for another. For example, a demonstration that no change in behavior occurred in the depletion task over time but carry-over effects did emerge, would present strong disconfirming evidence for the strength model (Baumeister et al., 1998, 2007). On the other hand such an outcome would not be problematic for models that attribute depletion effects to task switching aspects of cognitive control, where it is the nature of the two tasks that is important, not what happens within each task (Dang et al., 2013, 2017). Moreover, examining what happens in the depletion task is important from a methodological perspective. Most experiments employ a “hard” version of the depletion task in the experimental group (crossing out letters according to a complex set of rules) and an “easy” version of the task in the control group (crossing out every letter) without ever determining (a) that the hard version produces decrements in performance, (b) that the simple version does not produce decrements in performance, and, most importantly, (c) that decrements in performance on the hard task are more substantial than in the easy version of the task. However, if performance deteriorated in similar ways in both hard and easy versions of the depletion task, then much of the literature that has been cited to invalidate the strength model would itself be called into question if there were no differences between experimental and control groups on the depletion task. Lastly, the Dang et al. (2013) demonstration that people can adapt to the depletion tasks suggests the possibility of individual differences in depletion. If this were the case, it might be possible to identify a group of participants for whom depletion effects are minimal and a group who show severe depletion. From a strength model, carry-over effects would expected to be more pronounced for the second group than the first. We assert that much of the ambiguity of regarding self-regulation research could be eliminated if performance on the depletion task was monitored over time.

In the absence of a consensus about what tasks are actually depleting, we propose an empirical approach to assessing the importance of the above factors in inducing depletion both within the depletion task itself, and carryover effects on an outcome task. One approach to examining depletion is to track performance on the depleting task as it is being completed. If the task exhausts self-control resources then a testable consequence will be an observable decline in performance. The absence of any decline would be a clear indicator that the task does not induce depletion or participants can adapt to the task and replenish the resources (Dang et al., 2013). The observation of declining performance would provide *prima facie* evidence that depletion could be occurring. The first aim of the current experiments was to test this fundamental assumption of the strength model of ego depletion by measuring performance over time on a commonly-used depletion task. The second aim involved evaluating the claims that Baumeister and Vohs (2016) made regarding the conditions for depletion to be induced, specifically the need for a habit-forming stage and the effects of presentation modality, response modality time on task, and degree of task exposure. Thirdly, we test the utility of individual differences in depletion effects as a further means of testing the assumptions of the strength model.

One of the most commonly-used depletion induction activities is a letter crossing task (Hagger et al., 2010). This task requires participants to scan and identify words within text containing a target letter (commonly the letter “e”) and then identify words where the presence of the letter satisfies a set of conditions. We chose one of the simplest versions of the letter-crossing task similar to the one used by Hagger and Chatzisarantis (2016) that did not involve any pre habit-formation. By not including habit formation, we test a central claim of Baumeister and Vohs (2016) that depletion effects are not induced unless there is a habit-forming stage. One unlikely but possible interpretation of this claim, is that performance on the depletion task might not deteriorate across time, since self-regulation is not required. Alternatively, there might be deterioration across the task of some cognitive resources, but since self-control resources are not depleted, carry-over effects on an outcome task would not be expected. Finding such carry-over effects when there was no habit formation, when the stimuli were presented on a computer, and no motor response was required, would invalidate most of Baumeister and Vohs' claims.

The basic stimuli were held constant across the five studies and involved five short passages of text that varied in length between 150 and 400 words. The first study adopted a paper-pencil letter-crossing procedure using hand-written passages that the participant completed for 10 min (the most commonly used length of time for the letter-crossing task). In study two, the same stimuli and procedures were used but participants were not timed, rather they completed the full task regardless of how long it took. To examine whether computer administration would produce performance changes, in study three participants were once again not timed but were presented with the stories on a computer screen, and participants verbally identified target items. In study four participants were presented with a fully online version of the task where the stories were again presented on a single screen and participants were required to click on the actual letter “e” in the target word. In this version, the depletion task was restricted to 10 min. Study five was conducted to again document depletion over time, but to also confirm that depletion effects on the letter-e task had carryover effects on a working memory task. It also introduced an individual differences approach to understanding depletion effects where we compare performance of people who do not show changes in performance across the letter-e task to those who do show deteriorating performance across time.

Given Lurquin and Miyake (2017) comments regarding operational definitions of self-control, proponents of a strength model could argue that doing the letter-e task involves a series of steps, even without a habit forming stage. Detecting an e in a word is the first step in response chain. Having detected an e, participants need to compare surrounding letters and either inhibit a circling response if the letter is not surrounded by a vowel, or proceed to the next decision point if there is a neighboring vowel. Having determined that one of the surrounding letters is a vowel, the third step involves either proceeding with a response or inhibiting that response. The decision to not respond at two points in the process involves self-control. Each decision to not respond should deplete self-control resources such that participant scores on identifying target letters should decrease with increasing exposure to the task. Thus, from the perspective of the strength model, it is hypothesized that target detection should deteriorate across time on the letter-e task, and to the extent that this happens, this should produce carry-over effects on a subsequent outcome task.

## Study 1

This study commenced the study of depletion effects in time by presenting the Letter-e task in a pencil and paper format. The five stories were presented across 13 pages of handwritten text, with each story being written by a different person.

### Methods

#### Ethics statement

The following studies were approved by the Human Research Ethics Committee at the host institution (H15REA055). Written informed consent was obtained from all participants in Studies 1 to 3, and Study 5. In Study 4, participants consented via entering a unique code into a consent field on the computer page.

#### Participants

Study one included a mixed sample of 111 students and community residents (40.7% women). The average age of the sample was 29.04 years (range = 15–64, *SD* = 11.15; one participant did not correctly enter their age).

#### Tasks and scoring

The first version of the letter “e” task used five short stories sourced from the internet. These were first transcribed by hand onto 13 pages of lined paper and then photocopied for each participant. While there were differences in hand writing across stories, all were legible enough that participants could identify target items.

The same set of instructions was administered across all five studies, with modifications on how to produce a response (circle a word, say the word, click on the e). The initial instructions indicated that the task was challenging and required attention to detail. It gave a general overview of the task that indicated the requirement to find a specific target letter, “e,” among text words. The instructions then introduced the two rules that participants had to adhere to. Rule one required the “e” to be followed or preceded by another vowel in which case the word was circled. Rule two required that if the accompanying vowel was an “i,” the participant did not circle the word. The participants were then given some practice applying the rules to ensure that they understood the task requirements. The final instruction was to work as quickly as possible without making errors.

In this study the experimenter timed the participants and the task was restricted to 10 min. For each participant, the proportion of target words correctly detected on each page was measured.

### Results and discussion

**Figure 1:**
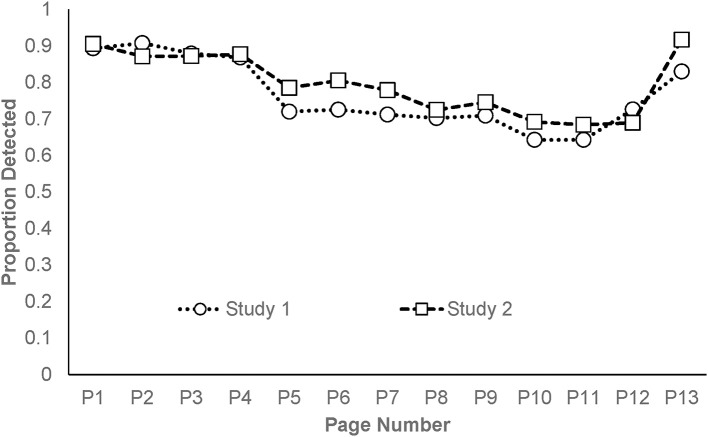
Proportion of targets detected as a function of page number in Studies 1 and 2.

**Figure 2:**
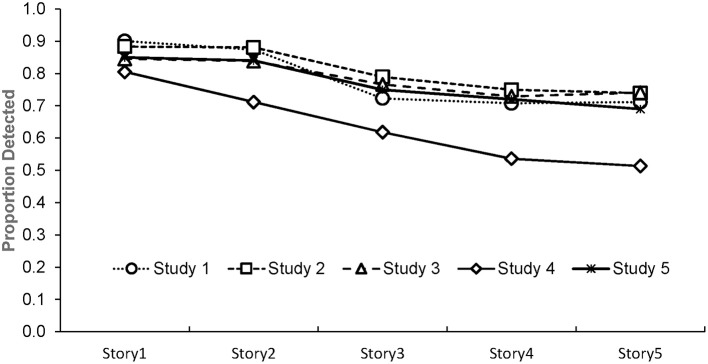
Proportion of targets detected as a function of story number in Studies 1–5.

Data for all five studies are available via the Open Science Framework (https://osf.io/5fhvm/). The data report accuracy across time as a function of the stories (1 through 5) and pages (1 through 13). The mean proportion of words correctly identified per page are summarized in Figure 1, and at the story level in Figure 2. It is clear that there is a deterioration in performance with time until the last page where there is an improvement on the task, consistent with the proposal that participants can conserve resources for a final push when the end point is known (Baumeister, 2001). Excluding the final page, 75% of the variance in target identification across pages is accounted for by a linear function. Because the time period for doing the depletion task was fixed, the number of data points contributing to the average varies by page. For this reason, accuracy from the first two pages, the last two pages completed (which varied depending on how many pages each participant finished), and an average of the pages in between, were compared for the 102 participants who met the threshold of completing at least five pages. For those that completed all 13 pages, the scores on page 12 were used as their final page. The mean proportion of targets correctly detected on each of these five “pages” in order were 0.89 (*SD* = 0.13), 0.90 (*SD* = 0.13), 0.82 (*SD* = 0.14), 0.74 (*SD* = 0.24), and 0.59 (*SD* = 0.30). A repeated measures ANOVA indicated there was a large and significant difference between the five page-groupings, *F*_(4, 404)_ = 57.89, *p* < 0.001, η_*p*_^2^ = 0.36. *Post-hoc* tests indicated that there was no significant difference between pages 1 and 2, but these were significantly different from the remaining pages, which all differed from each other (*p* < 0.05). The difference between the first and last pages produced a large standardized effect size of Cohen's *d* = 1.03.

## Study 2

Study two was conducted to examine the extent to which results from Study 1 were merely an artifact of the artificial time constraints placed on the letter-crossing task. While the time constraint was imposed to ensure the tasks mirrored the method of implementation in prior experiments, we sought to determine whether the pattern of performance deterioration would be replicated after removing this constraint.

### Methods

#### Participants

Study two recruited a sample of 20 community participants (50% women). The average age of the sample was 34.5 years (range = 22–60, *SD* = 11.82).

#### Task and scoring

The same letter crossing task as in Study 1 was used, however, participants were required to complete all 13 pages. There was no time limit on the duration of the task and completion times were not recorded.

### Results and discussion

As with Study 1, the mean proportion of words correctly identified per page are summarized in Figure 1, and at the story level in Figure 2. As is evident in the figures, the pattern of results of this study are virtually identical to those obtained in Study 1. Again, when performance is plotted as a function of page number, there is a strong deterioration in performance across time, until the final page where performance again improves. A one-way repeated measures ANOVA indicated that there was a large and significant omnibus difference between the 13 pages, *F*_(12, 228)_ = 5.85, *p* < 0.001, η_*p*_^2^ = 0.24. Ignoring the apparently anomalous page 13, the linear decrease across pages accounted for 93% of the variance in accuracy across pages and performance significantly deteriorated from initial levels by page five. The difference between the first and last pages once again produced a large effect (*d* = 0.84).

## Study 3

Typically, the letter crossing task has been presented in pencil and paper format, however, the task has also been presented in computer format in the laboratory (Schmeichel, 2007; Schmeichel and Vohs, 2009) and via the internet outside the laboratory (e.g., 23) with mixed results. While Schmeichel and Vohs (2009) reported reduced pain tolerance in ego-depleted participants (and thus, success of the depletion measure was inferred), Allmond (2013) found no difference between participants exposed to the depletion and control conditions on subsequent measures of self-control. Central among Baumeister and Vohs (2016) criticisms of the failed multi-site replication was the assertion that the use of computer-administered tasks was sub-optimal for inducing and testing depletion. Given the benefits of conducting research electronically and outside of a laboratory setting (e.g., increasing external validity; reduced cost), the remaining studies were interested in whether depletion would be evident in electronic versions of the task.

Study three was conducted to examine the effect of variations in task administration and participant response procedure. Specifically, the task used computer administration and verbal response instead of the paper-pencil administration and response used in Studies 1 and 2.

### Methods

#### Participants

Study three recruited 115 community-based volunteers (56.52% women). The average age of the sample was 36.61 years (range = 18 to 78, *SD* = 16.39).

#### Task and scoring

In this study the depletion task was presented on a computer by way of a Microsoft PowerPoint program. Each story was presented on a single slide in a Time New Roman 10.5 font and participants were required to complete all five stories (slides). In this instance, the participants identified each target word by saying it aloud. The experimenter recorded the number of correct responses (and errors) on each story as well as the time it took to complete each slide.

### Results and discussion

Accuracy over time is plotted as a function of the story in Figure 2. Again, performance deteriorates from the first story to the fifth story. The linear decrease across stories accounted for 85% of the variance, but a cubic function accounted for 99% of the variance. A one-way repeated measures ANOVA indicated that there was a large and significant omnibus difference across the five stories, *F*_(4, 456)_ = 32.73, *p* < 0.001, η_*p*_^2^ = 0.22. Linear comparisons showed that accuracy between Story 1 and 2 did not differ. However, accuracy for Stories 3, 4, and 5 were significantly worse than Stories 1 and 2. Stories 4 and 5 did not differ significantly from each other. The difference between the first and last stories produced a medium effect size of *d* = 0.61.

## Study 4

Study three was conducted to replicate the effects of computer-administration identified in Study 3, while further varying the task parameters to increase the complexity of the task, the potential variability in accuracy (increasing target number), and the response procedure.

### Methods

#### Participants

The sample consisted of 256 community-based volunteers (57.8% women). The average age of the sample was 37.52 years (*SD* = 15.56).

#### Task and scoring

In this study the depletion task was again presented on a computer, but this time using a purpose-built program. As was the case in Study 3, each story was presented in its entirety on a single screen. The response format differed in that participants had to use the computer mouse to “click” on every target “e.” This added more targets and an additional element of complexity to the task. If the target word was “true,” the participant would click on the “e.” However, if the target word was “feel” the participant had to click on the first “e” and click on the second “e.” The participant only clicked on “e”s within the vowel pair; if the target was “learned,” they would only click on the first “e” and not on the second. Given Study 1 and 2 revealed that having a timed administration did not change the pattern of results, for Study 4, participants did the depletion task for a fixed period of 10 min.

### Results and discussion

The results of the study are summarized at the story level in Figure 2. There was a clear deterioration across the five stories with the linear component accounting for 97% of the variance. As was the case in Study 1, the time period for doing the depletion task was fixed so the number of data points differs for each of the stories. To conduct statistical analysis of the data, the current story data were sub-divided into the same page groupings as used in Study 1. Thus, the first two pages, the last two pages, and an average of the pages in between, were analyzed for the 211 participants who completed at least five pages. The mean proportion of targets detected on each of the five “pages” in order were 0.76 (*SD* = 0.18), 0.87 (*SD* = 0.17), 0.68 (*SD* = 0.18), 0.63 (*SD* = 0.21), and 0.62 (*SD* = 0.30). A one-way repeated measures ANOVA indicated there was a large and significant omnibus difference between the five conditions, *F*_(4, 844)_ = 74.70, *p* < 0.001, η_*p*_^2^ = 0.26. Performance on the first two pages was significantly different to the last two pages, confirming a deterioration in performance and the difference between the first and last pages produced a medium effect size of *d* = 0.53.

## Study 5

Studies 1–4 have shown that performance deteriorates across time on the letter-e task and the decrement is present with changes in presentation modality and response modality. The results are at odds with some of Baumeister and Vohs (2016) claims regarding the conditions under which depletion effects will be observed. While we have shown a decrement across time on one measure of the letter-e task, it is not clear what this decrement is reflecting Boredom, physical fatigue, changes in mood, lack of motivation, or depletion of some other cognitive resource are plausible alternative explanations for decrements in task performance. In addition, while we have shown that performance deteriorations are largely invariant across several task formats, we have not tested the critical assumption of the strength model that these decrements transfer to outcome tasks, and we have not tested the Baumeister and Vohs claim that a habit forming stage is necessary for carry-over effects to be observed.

In Study 5, we address these issues by first introducing an individual differences approach to explore performance on the letter-e task, and then adopt a similar methodology to explore carry-over effects. Here we adopt a distinction made by Healey et al. (2011) regarding extrinsic and intrinsic influences on task performance. Extrinsic factors are those that are external to the cognitive system under consideration, whereas intrinsic factors reflect internal changes to the cognitive system itself. Thus, comparing performance of participants who have to respond or not respond on a Letter-e task under a complex set of requirements, to participants who simply have to identify any letter e that they encounter, would reflect, external factors. However, the strength model assumes that performing the letter-e task produces internal intrinsic changes to the self-regulatory system, in that self-control resources become depleted. Moreover, as Healey et al. (2011) argue, even within a group that has been exposed to a demanding task there is going to be individual variation in some fundamental aspect of the relevant system, such that participants perform the task better than others, perhaps due to a better ability to control or regulate self-control resources. Thus, an individual difference approach to intrinsic depletion effects appears to be an alternative, and perhaps more appropriate, methodology for examining ego-depletion than the typical extrinsic control group vs. experimental group methodology that dominates current practice.

In the extensive literature that addresses individual differences in executive functions and working memory, two basic methodological traditions have emerged, a latent-variable analysis approach and an extreme-groups approach (Friedman and Miyake, 2004; Conway et al., 2005). For example, in many of studies adopting the extreme-group approach to individual differences in working memory capacity, participants are first tested on the operation span task, or similar complex span tasks. In the operation span task participants are presented with a series of maths problems, each of which is paired with a word. For each pair, the task is to process the maths problem and remember the word. At the end of a sequence of such pairs, participants are required to recall the words in the order in which they were presented. It is performance on the memory component that is indicative of working memory capacity.

Following testing on the operation span task, participants are subsequently divided into high (upper quartile) and low (bottom quartile) working memory capacity groups. Differences between the high and low capacity groups on other cognitive tasks (e.g., Raven's progressive matrices, antisaccade tasks, Stroop task, etc.) has been taken as evidence for the involvement of working memory in those tasks. Using this methodology working memory is implicated in higher-order cognitive task such as, comprehension, reasoning, and problem solving (Engle, 2002), in fluid intelligence (Unsworth et al., 2009), and in more elemental cognitive processes such as, attentional control, interference resolution, and resistance to distraction (Engle, 2002).

In the absence of any independent evidence that the letter-e task measures self-regulation resources, we first test the possibility that individual differences in working memory capacity, rather than self-regulatory capacity, underpin the letter-e task. To this end, the operations span task was administered prior to the letter-e task. High and low WMC groups were subsequently identified and performance on the letter-e task was then compared across these two groups.

Carry-over effects of the Letter-e task were also examined in the context of the operation span task, a task that has been previously shown to be sensitive to negative transfer effects (Healey et al., 2011). In administering the operation span task a second time after completion of the letter-e task, we have pre-test and post-test measures on the operation span task that permits the evaluation of any intrinsic changes in the self-regulatory system that result from engagement with the depletion task. There is some existing evidence that there is individual variability on the depletion task. Dang et al. (2013) showed that participants can habituate to the depletion task, such that carry-over depletion effects were absent in the adaptation group. This finding suggests the possibility that some participants may adapt to the letter-e task and not deplete self-control resources. If such a group did exist, a clear prediction of the strength model is that this group of participants should not show carry-over depletion effects.

The adoption of a pre-test/post-test design on the operation span task introduces the complication of practice effects. Prior research with large N's (80–300) examining the test-retest reliability of the operation span task, has shown correlations around 0.70 for pre-test and post-test memory measures with performance significantly improving by 2 to 3 items from first to second testing (Redick et al., 2012; Gonthier et al., 2016). Thus, a post-test advantage would normally be expected under conditions where there is no depletion. Given that in other domains, deficits in executive function are associated with the absence of or weaker repetition effects (Darby et al., 2002; Duff et al., 2012), it is plausible that intrinsic carry-over depletion effects might be reflected in absent or weakened repetition effects. A strong test of the strength model would be reflected in significant repetition effects for the group who show no depletion effects on the letter-e task. Weak or absent repetition effects would be expected for the group who do show depletion effects on the letter-e task.

### Methods

#### Participants

Eighty-seven adults volunteered to participate in the experiment. Of these, 51% were women and the average age of the participants was 41.2 (*SD* = 19.8) years. Of the total number of participants, 58 were tested on both operation span and letter-e tasks. A group of 29 were only tested on operation span in order to confirm that repetition effects were present under our experimental conditions.

#### Materials

The operation span task involved the participants being administered eight four-pair trials. Each trial in the task involves four pairs of simple arithmetic problems (e.g., 4/2 + 1 = 3) followed by a word (e.g., “cloud”). Each maths problem was presented on the screen and the participant had 4 s to decide if the provided answer was correct. After the maths problem disappeared from the screen a word appeared for 1 s. The participant was required to say the word out loud and try to remember it. After four such pairs, a row of question marks appeared as the cue for the participants two write down the four words in the order they had been presented in. The response sheet contained spaces for four responses on each trial and instructions stressed recall from the first word leaving blank spaces for any forgotten words. Participants were told the task was a difficult one and they were to make sure they got the maths problems correct and then do their best to remember the words.

#### Procedure

The experimental session started with the first administration of the operation span task. Following this, those in the depletion condition completed the letter-e task as described in Study 3. The second administration of the operation span task using a different set of materials then followed.

In order to confirm that repetition effects on the operation span were present in our study and of much the same magnitude as in previous studies, a group of 29 participants were tested twice on the operation span task with a 15-min interval between tests. The experimenter engaged the participant in conversation across this period. A repeated measures *t*-test confirmed that memory performance improved from initial test (*M* = 23.45, *SD* = 5.36) to post-test (*M* = 26.13, SD = 4.39), *t*_(28)_ = 4.34, *p* < 0.001, *d* = 0.53. The difference of 2.69 items is consistent with that obtained in large N studies (Redick et al., 2012; Gonthier et al., 2016).

### Results and discussion

Figure 2 shows that the pattern of scores on the letter-e task mirrored that of the earlier studies when participants in the experimental group were considered as a whole. Performance deteriorated with time on task, *F*_(4, 228)_ = 24.01, *p* < 0.001, η_*p*_^2^ = 0.29. Significant failures in detection had emerged by the third story (*p* < 0.001) and continued to decline (Cohen's *d* = 0.93 for the difference between Story 1 and 5). The linear decrease across stories accounted for 94% of the variance, but a cubic function accounted for 97% of the variance.

#### Working memory capacity

**Figure 3:**
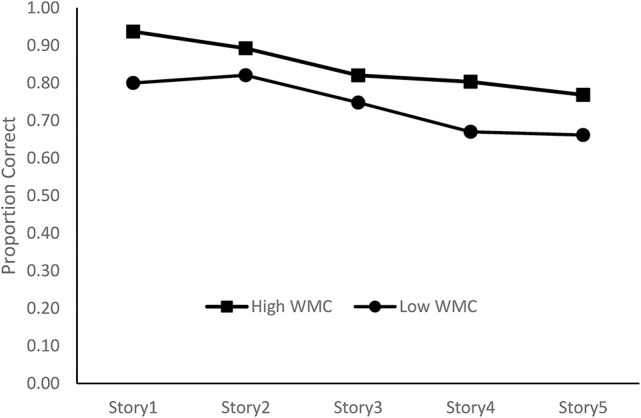
Proportion of targets detected as a function of working memory capacity group.

Following standard practices in extreme-group analyses, performance on the pre-test memory results of the operation span task was used to construct a High WMC group (*N* = 15, M_correct_ = 92%) and a Low WMC group (*N* = 15, M_correct_ = 42%). The performance of these two groups on the Letter-e task is presented in Figure 3, and was analyzed by a 2 group by 5 stories mixed-design ANOVA. Performance deteriorated across the five stories, *F*_(4, 112)_ = 13.47, *p* < 0.001, η_*p*_^2^ = 0.32, those in the High WMC group were more accurate in detecting target items than the Low WMC group, *F*_(1, 28)_ = 6.85, *p* = 0.014, η_*p*_^2^ = 0.20. Importantly, the rate of deterioration was the same for both groups in that there was no significant interaction, *F*_(1, 112)_ = 0.70, *p* = 0.591, η_*p*_^2^ = 0.03. According to the logic of the extreme-groups design, working memory capacity is related to the accuracy of target identification, but not related to the decline in performance across stories.

The carry-over effects onto post-test memory performance were also examined. Performance increased from pre-test (*M* = 21.50, *SD* = 8.61) to post-test (*M* = 22.47, *SD* = 8.73), *F*_(1, 28)_ = 5.19, *p* = 0.031, η_*p*_^2^ = 0.16, and there was no difference in the strength of the repetition effect for each group, *F*_(1, 28)_ = 1.78, *p* = 0.193, η_*p*_^2^ = 0.06. However, the average improvement of 0.97 items, is less than that found under conditions where the letter-e task did not occur between test sessions. In sum, completing the letter-e task did reduce the magnitude of repetition benefits, but there were no differential effects on either the letter-e task or repetition effects on the operation span task as a function of working memory capacity.

#### Adaptation to the letter-e task

**Figure 4:**
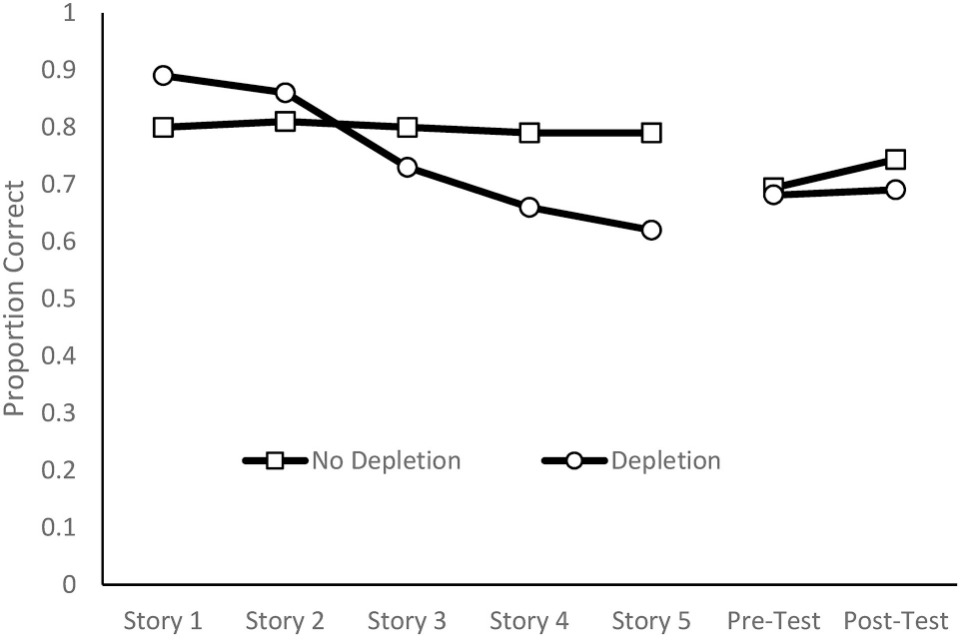
Proportion of targets detected and words remembered as a function of depletion group.

The extreme-groups approach was adapted to explore intrinsic carry-over effects between the Letter-e task and the operation span task, with the degree of deterioration on the Letter-e task being the dimension for group selection. The linear slopes reflecting changes in performance across the five stories were calculated for each person. The slope values ranged from 0.06 to −0.12, with positive slopes indicating improvement across stories and negative slopes indicating deteriorating performance across stories. Twenty-six participants who has slope values between 0.06 and −0.03 was assigned to the no-depletion group. A repeated measures ANOVA or this group indicated that there was no significant difference in target detection across the five stories, *F*_(4, 100)_ = 0.20, *p* = 0.939, η_*p*_^2^ = 0.01. Figure 4 clearly shows the same level of performance on each of the stories. The remaining thirty participants who had slope values of between −0.04 and −0.12 constituted the depletion group. Figure 4 shows a consistent deterioration in performance across the five stories. A repeated measures ANOVA confirmed that there was a significant deterioration across stories, *F*_(4, 124)_ = 49.29, *p* < 0.001, η_*p*_^2^ = 0.61.

Figure 4 and Table 1 shows performance on the critical memory component. For the no depletion group, performance improved by 1.5 items from pre-test to post-test, *t*_(25)_ = 2.76, *p* = 0.011, *d* = 0.28. For the depletion group there was no significant improvement, *t*_(31)_ = 0.49, *p* = 0.624, *d* = 0.04. The no depletion group conceptually replicates the Dang et al. (2013) outcomes in that those who adapted to the depletion task did not show depletion effects. In contrast, the participants who did show decrements in performance on the letter-e task, an outcome that is consistent with depletion assumptions, did not show any improvement from pre-test to post-test. One way of explain this outcome is to assume that the benefits of repetition have been offset by depleted resources.

**Table 1:** Mean levels of performance (SD) on the operation span task for depletion and no depletion groups.

		**Experimental**	
		**No depletion**	**Depletion**
Maths problems	Pre-test	28.00 (4.98)	27.47 (5.83)
	Post-test	29.11 (3.48)	27.43 (6.60)
Memory recall	Pre-test	22.71 (5.82)	21.33 (6.98)
	Post-test	24.12 (5.60)	21.70 (7.81)

## General discussion

The current experiments are based on the conviction that self-control research using the sequential task paradigm must examine performance on the depleting task if current debates are to be resolved. The studies were designed to test the assumption that, under a limited set of conditions (Baumeister and Vohs, 2016), extended performance on an executive function task would lead to depletion of self-regulatory resources. Surprisingly, changes in performance on the depletion task are rarely empirically tested but should be, given Dang et al. (2013) demonstration that participants can adapt to the depletion task. Therefore, the current studies were designed to first determine whether performance declined over time in a task similar to that used in the multi-site replication trial (Hagger and Chatzisarantis, 2016), and, secondly, in response to Baumeister and Vohs (2016) assertions, to determine how performance changed with changes to presentation and response formats. The third objective was to introduce an individual differences approach to address the nature of the letter-e task, and to understand carry-over effects from the letter-e task.

The first two objectives were achieved across five independent studies that measured performance on the letter canceling task in which we varied the format of the studies. In general, the results of all studies indicated that correct detection of target items deteriorated across time. The trend over time yielded very large effect sizes (η_*p*_^2^ > 0.22) and pre-post (performance between first vs. last stories) effects were medium to large and consistent with the pattern expected under ego-depletion (see Table 2 for a summary). In order to compare performance across the five studies, we used a meta-analysis methodology to evaluate the importance of the changes in presentation and response modalities across the studies. In this analysis we were more interested in the heterogeneity of the experimental procedures rather than the overall effect size. Our initial analysis, using a random effects model, indicated that there was significant heterogeneity among the studies, and there was a small-study bias as reflected in a significant correlation between effect size and standard error of the mean. When Study 2 was omitted, the average Hedges' *g* effect size of −0.79 was significantly different to zero, 95% CI [−0.95, −0.64], *p* < 0.001, heterogeneity was not significant, *Q*(3) = 5.49.6, *p* = 0.14, *I*^2^ = 45.41, and there was no small-study bias. Our results thus confirm that the letter-canceling task is one that is appropriate for use in ego-depletion studies in that performance decrements do emerge over time and that presentation and response modalities are not crucial determinants of the size of the effect. Whether the materials were presented in handwriting, or on computer screen using type face of whole stories, or words presented individually, did not particularly matter in producing decrements in performance. Neither did response modality of producing a written response, a verbal response or a mouse click produce differential impacts. In short, we failed to find support for Baumeister and Vohs (2016) claim that computerized methods are inappropriate for producing depletion effects. In fact, the computerized version of the task used in Study 4 produced the largest absolute decrement in performance.

**Table 2:** Summary of study characteristics and effect sizes.

**Study**	**Presentation**	**Response**	**Time**	**Accuracy decline (%)**	**All pages η*_*p*_*^2^**	**1st vs. last page/slide Cohen's *d***
1	Handwritten hardcopy	Paper-pencil	Timed	20	0.36	1.03
2	Handwritten hardcopy	Paper-pencil	Untimed	16	0.24	1.09
3	Computer screen	Verbal	Untimed	12	0.22	0.66
4	Computer screen	Mouse Click	Timed	36	0.26	0.55
5	Computer screen	Verbal	Untimed	19	0.29	0.83

The third objective regarding carry-over effects confirmed the necessity to measure performance on the depletion task. By introducing a pre-test post-test design using the operation span task as an outcome task we were first able to provide some independent assessment of what cognitive resources underpin the letter-e task. Specifically, we were able to show that high working memory capacity resulted in more accurate performance on the letter-e task, but the rate of decline of performance was independent of differences in capacity. By applying the same basic individual differences approach to carry-over effects between the letter-e task and the operation span task, we were able to confirm Dang et al. (2013) finding that some participants' performance did not deteriorate across time. Those participants did not show any carry-over depletion effects on the working memory task. Presumably, these participants were able to adapt to the letter-e task and replenish or regulate resources throughout the duration of the task (Dang et al., 2013). For those, who did show deterioration on the letter-e task group repetition effects were absent, indicative of carry-over depletion effects.

## Theoretical implications

The research questions were framed around the strength model of ego-depletion. Two aspects of the current research were consistent with that model. Firstly, performance on the depletion task did deteriorate across time when averaged across participants. Secondly, those who did show depletion effects on the letter-e task did show carry-over effects on the working memory task, whereas those who did not show any evidence of depletion did not exhibit carry-over effects either. Thus, the strength model survived two tests where it could conceivably have failed.

Having said that carry-over effects were consistent with the strength model, we would issue a caveat. Lurquin and Miyake (2017) raise the point that the current ego-depletion studies suffer from a lack of specificity regarding how self-control is operationalized in both depletion and outcome tasks. We have argued that our version of the letter-e task involves self-control by the requirement to inhibit responses under specified circumstances. We have not addressed how these same inhibitory responses are involved in the operation span task, and we do not think that is possible to do so in any straightforward manner. Thus, if one assumes, as does the strength model, that the same resources must be involved in both tasks, carry-over effects from a task where inhibitory processes are involved to a task where such processes are not obviously involved is not consistent with the model.

Given the general acknowledgement that the operation span task reflects working memory capacity, it is possible to account for the current data without reference to self-control. Our results show that individual differences in WMC are related to accuracy on the letter-e task, but not to the rate of decline across stories. Thus, working memory resources underpin the task. By dividing groups on the basis of slopes, we have in effect identified groups of participants within each WMC group who appear to be able to regulate WMC throughout the letter-e task resulting in normal repetition effects on the operation span task, and a group that shows depletion of those resources that carryover to the absence of repletion effects. That these participants come from both high and low WMC groups is evident that there are no differences on pre-test operation span performance. In short, an alternative explanation for the current outcomes is that the letter-e task depletes working memory resources for some individuals, but others are able to conserve or regulate these resources. Importantly, form this perspective no assumptions need to be made about how self-control is operationalised in either task.

However, there was one other aspect of the results that was clearly not consistent with the strength model. Our findings do not support the claim by Baumeister and Vohs (2016) that habit-formation is necessary for producing depletion. We show carry-over depletion effects where no habit-forming stage has been included in the depletion protocol. However, it remains an open question whether this habit-formation would enhance the depletion effect and this is an important area for future investigation. Be that as it may, our results suggest that the reason for failed replication by Hagger and Chatzisarantis (2016) cannot be attributed to the way in which the depletion task was administered.

Our results also have implications for other theoretical accounts. The process model proposed by Inzlicht and Schmeichel (2012) explains self-regulation problems in terms of the interplay between motivation and attention. They propose that during the depletion task there is a change in motivation away from exerting control to acting on impulse. With the change in motivation there is a corresponding move in attention away from the cues that signal that control is required toward cues that signal reward. To the extent that our target detection measure reflects changes in sensitivity to the cues for control, the current results would suggest that there is a gradual change in motivation across time, another intrinsic factor, and that some participants are able to maintain motivation and others not. The results do question the usefulness of taking a single measure of motivation after the depletion task has been completed, as is common practice. Rather, there is a pressing need for a measure of motivation that can be administered repeatedly in order to demonstrate that the changes in sensitivity to control cues over time is mirrored in motivational changes.

The outcomes are potentially consistent with explanations based on task switching (Dang et al., 2013) or goal maintenance (Dang et al., 2017). In these models, the carry-over depletion effects are attributed to the cost associated with switching or maintaining control processes across tasks or with maintaining goals throughout the depletion task. Thus, those who are able to maintain goals throughout the depletion task are less likely to show carry-over effects than those who are less able to maintain the goal of detecting and reporting specific instances that conform to a nominated rule. Goal maintenance is unlikely to be affected by presentation or response modalities.

Our demonstration of individual differences on the depletion task and their subsequent effect on outcome tasks has clear implications for prior meta-analyses. Without knowing what proportion of participants adapt to the depletion task in each study, the true carry-over effect size of those who were depleted may well be an underestimated in those studies.

## Methodological implications

Our results demonstrate the need to carefully examine performance on the depletion task. We have shown that there are individual differences on the task and that such differences have implications for whether or not carry-over effects are or are not observed. We have shown that, at least for the letter-e task, reliable changes in behavior can be detected, and that such changes, when they occur, are related to changes on a subsequent outcome task. We have shown that decrements in performance can be obtained across different presentation and response conditions and that rapid deterioration in behavior is not necessarily associated with a fixed period or a fixed workload or related to sample size. In all cases, depletion effects were observed and the magnitude of effect did not vary systematically with sample size. Thus, the first contribution of the current experiments is that we start the discussion of what task parameters are required to produce carry-over effects.

There are several things we have not demonstrated that may emerge as being critical. While we have adopted target detection as our measure of depletion and have shown that this measure is relevant to the observance of carry-over effects, it is not clear if indeed it is the most pertinent measure of performance. For example, from a goal maintenance perspective, our version of the letter-e task requires that participants keep three goals in mind: they have to detect all e's, then reject an e if it is not followed or preceded by a vowel, and then to respond only when that second vowel is an a, e, o, or u. Failures to maintain any of the three goals could lead to non-detection of the target. The implication being that a control or “easy” version of the letter-e task, where people only have to detect every letter e, could in itself be depleting. Many of the current failures to show depletion effects between experimental and control groups might be due to depletion effects in the control condition.

We have suggested that working memory capacity depletes across time and that target detection is related to working memory capacity and consequently could be a questionable measure of self-regulatory failure. The letter-e task allows more direct tests of goal maintenance and self-regulation failures, in that while given instructions not to respond to *ei* or *ie* words, some participants do produce these errors. Given that these are clear failures to follow instructions, it might prove that these errors are a better means of testing the assumptions of the strength model. Moreover, it may be the case that the commission of these errors are related to individual differences in completion time, or accuracy of target detection, the number of pages completed, or changes in motivation. This clearly reinforces Lurquin and Miyake (2017) need for experimenters to operationally define how self-regulation is operationalised within a given depletion and outcome task. As such one clear line of future research involves the best way to explore the relationship between target detection and false alarms with other characteristics of the depletion task and then to determine how these factors influence any subsequent carry-over effects.

Past research in ego-depletion has explored the breadth of ego-depletion rather than empirically-derived explanations of the depletion process itself (Inzlicht and Schmeichel, 2012). It is thus not surprising that many authors have indicated the pressing need to examine performance on the depletion task itself (Inzlicht and Schmeichel, 2012; Inzlicht and Berkman, 2015; Lurquin and Miyake, 2017). The evidence presented here demonstrates that observable deterioration in target detection occurs over time in a letter crossing task that results in carry-over effects on a working memory task. On average, the effects we observed on the letter canceling task were medium to large and appear not to be dependent on habit-formation or the mode of administration and response. Individual differences on the depletion task and subsequent carry-over effects, confirm the need to examine performance on the depletion task in all experiments in both experimental and control conditions. The exploration of such individual differences based on theory-driven measures of self-control would substantially inform our theoretical understanding of the depletion process. With regards to the assumptions of strength model, the data were consistent with some of the assumptions but not all. Moreover, the results of Study 5 suggest that the results can be accounted without recourse to a role for self-regulation resources.

## Ethics statement

This study was carried out in accordance with the recommendations of NHMRC National Statement on Ethical Conduct in Human Research (2007) that governs research involving human participants in Australia. All subjects gave written informed consent in accordance with the Declaration of Helsinki. The protocol was approved by the Human Ethics Committee of the University of Southern Queensland.

## Author contributions

All authors were involved in the conceptualization and design of at least one experiment. Data acquisition: MA, RF, and JT. Analysis, MA, GT. Interpretation: All authors. Initial drafting: MA and GT. Revising: all authors. Final approval has been given by all authors and all have accepted accountability for the work.

### Conflict of interest statement

The authors declare that the research was conducted in the absence of any commercial or financial relationships that could be construed as a potential conflict of interest.
